# The influence of neoadjuvant chemotherapy on complications of immediate DIEP flap breast reconstructions

**DOI:** 10.1007/s10549-019-05241-9

**Published:** 2019-04-27

**Authors:** J. Beugels, J. L. W. Meijvogel, S. M. H. Tuinder, V. C. G. Tjan-Heijnen, E. M. Heuts, A. Piatkowski, R. R. W. J. van der Hulst

**Affiliations:** 1 Department of Plastic, Reconstructive and Hand Surgery, Maastricht University Medical Center, P.O. Box 5800, 6202 AZ Maastricht, The Netherlands; 2 GROW – School for Oncology and Developmental Biology, Maastricht University, Maastricht, The Netherlands; 3 Department of Medical Oncology, Maastricht University Medical Center, Maastricht, The Netherlands; 4 Department of Surgery, Maastricht University Medical Center, Maastricht, The Netherlands

**Keywords:** Neoadjuvant chemotherapy, DIEP flap, Breast reconstruction, Free flap, Complications, Microsurgery

## Abstract

**Purpose:**

The impact of neoadjuvant chemotherapy on the surgical outcomes of immediate breast reconstruction remains controversial. The aim of this study was to analyze the incidence of complications of immediate deep inferior epigastric artery perforator (DIEP) flap breast reconstructions in patients who received neoadjuvant chemotherapy compared to patients without neoadjuvant chemotherapy prior to surgery.

**Methods:**

A multicenter, retrospective cohort study was conducted of all patients who underwent immediate DIEP flap breast reconstruction between January 2010 and June 2017. Patients were divided in two groups as breast reconstructions with or without neoadjuvant chemotherapy, respectively. The primary outcome was the incidence of postoperative flap re-explorations, recipient-site complications and donor-site complications.

**Results:**

In total 432 immediate DIEP flap breast reconstructions in 326 patients were included. Forty-eight patients (*n *= 67 flaps) received neoadjuvant chemotherapy prior to immediate breast reconstruction and 278 patients (*n *= 365 flaps) did not. No statistically significant differences for any major (4.5% vs. 10.4%; *p *= 0.175) or minor (16.4% vs. 24.7%; *p *= 0.191) recipient-site complication were observed. Donor-site complications were recorded in 9 (18.8%) and 62 (22.2%) patients, respectively (*p *= 0.587). There was no difference in need for flap re-exploration between groups (3.0% vs. 8.5%; *p *= 0.139). Correction for potential confounding variables did not result in significant differences.

**Conclusions:**

This study demonstrated similar complication rates for patients with and without neoadjuvant chemotherapy prior to immediate breast reconstruction, indicating that it is safe to perform an immediate DIEP flap breast reconstruction after neoadjuvant chemotherapy.

**Electronic supplementary material:**

The online version of this article (10.1007/s10549-019-05241-9) contains supplementary material, which is available to authorized users.

## Purpose

Neoadjuvant chemotherapy is increasingly being used for the treatment of patients with breast cancer. It is defined as systemic therapy that is administered prior to locoregional treatment. Despite its initial role in the management of locally advanced breast cancer, the indications have been extended and it is now routinely used in early stage breast cancer treatment as well [1–3]. The survival and distant disease progression rates after neoadjuvant chemotherapy have been shown to be similar to adjuvant chemotherapy [2, 3]; however, neoadjuvant chemotherapy is associated with a moderately increased local recurrence risk [3]. Neoadjuvant chemotherapy allows early evaluation of the response to therapy and improves tumor resectability by downstaging the tumor [2–5]. This allows more breast-conserving surgery, with up to one-third of patients eligible for breast-conserving surgery in whom mastectomy was initially indicated [3]. Hence, a mastectomy is still required in a considerable number of patients.

The postmastectomy reconstruction rates have increased over time and now trend toward more immediate bilateral implant-based breast reconstructions [6–8]. Nevertheless, autologous breast reconstructions provide a more natural and permanent result than implant-based reconstructions and offer higher patient satisfaction [9–11]. Of all options for autologous breast reconstruction, the deep inferior epigastric artery perforator (DIEP) flap is considered the first choice in most centers [12]. Immediate breast reconstruction, which yields better aesthetic results and may reduce psychological distress, can be offered to patients who are oncologically eligible [13, 14].

However, patients who receive neoadjuvant chemotherapy are less likely to undergo immediate reconstruction [15]. One of the reasons is that the impact of neoadjuvant chemotherapy on the surgical outcomes of immediate breast reconstructions remains controversial and highly variable results are reported in the literature [16–24]. In addition, all studies analyzed the total complication rates of either implant-based reconstructions [19], a combination of implant-based and autologous tissue reconstructions [21, 22], or a mix of several types of autologous reconstructions [16–18, 20, 24]. However, the complication rates are different for each type of breast reconstruction, regardless of the use of neoadjuvant chemotherapy; hence, they should be analyzed separately instead of being pooled. The aim of this study was to analyze the influence of neoadjuvant chemotherapy on the complication rates of immediate DIEP flap breast reconstructions only, by comparing the surgical outcomes of patients who received neoadjuvant chemotherapy to the outcomes of patients without neoadjuvant chemotherapy.

## Methods

A retrospective cohort study was conducted based on a prospectively maintained database of all patients who underwent immediate DIEP flap breast reconstruction at Maastricht University Medical Center in the Netherlands and two community hospitals, VieCuri Medical Center Venlo and Zuyderland Medical Center Sittard-Geleen, between January 2010 and June 2017. All patients who received neoadjuvant chemotherapy prior to immediate DIEP flap breast reconstruction were identified. The control group consisted of all patients who underwent immediate DIEP flap breast reconstruction without neoadjuvant chemotherapy. The study was approved by the institutional review board and was performed in accordance with the ethical standards of the Declaration of Helsinki.

Patients over 18 years old were considered eligible if they underwent immediate DIEP flap breast reconstruction with or without neoadjuvant chemotherapy. Patients with bilateral breast reconstructions who had unilateral breast cancer and a contralateral prophylactic or risk-reducing mastectomy were included, as well as patients who underwent bilateral risk-reducing mastectomy. However, patients with unilateral stacked DIEP flap breast reconstructions were excluded from this study due to the potential complications related to the complexity of the operation. All bilateral reconstructions were performed at the university hospital.

Neoadjuvant chemotherapy was offered to patients with stage III or early stage breast cancer when chemotherapy was already indicated by clinical stage and tumor characteristics. According to the hospital protocols, mastectomy and immediate breast reconstruction were planned approximately 5 weeks after the first day of the last chemotherapy cycle. Skin-sparing mastectomies were performed in all patients. Our surgical technique of DIEP flap breast reconstruction and the postoperative care has been published previously [25].

Patient characteristics (age, body mass index (BMI), comorbidities, smoking status), tumor characteristics, neoadjuvant chemotherapy regimens, time between the last chemotherapy cycle and surgery, adjuvant treatment, operative details (type of reconstruction, operative time, ischemia time), recipient- and donor-site complications, thromboembolic events, and flap re-explorations were recorded. Tumor characteristics included clinical TNM stage, histology, and hormone and HER2 receptor status. Complications were divided in major and minor complications. Major recipient-site complications were total flap loss, partial flap loss, and venous congestion. Minor recipient-site complications included infection, hematoma, seroma, fat necrosis, and wound problems. Minor donor-site complications were infection, hematoma, seroma, fat necrosis, wound problems, and bulging. Abdominal herniation confirmed by ultrasound was considered a major complication. Fat necrosis was defined as a palpable firmness identified by physical examination during postoperative evaluation or detected by ultrasound. Cases of partial flap loss were also registered as having fat necrosis. Pulmonary embolism and deep vein thrombosis (DVT) were considered as thromboembolic events. The follow-up duration was quantified as the time between the date of operation and the last visit to the outpatient clinic.

The primary outcome measure was the incidence of postoperative flap re-explorations, recipient-site complications, and donor-site complications following immediate DIEP flap breast reconstructions. Complication rates in patients who received neoadjuvant chemotherapy were compared to those of patients without neoadjuvant chemotherapy.

## Data analysis

Based on the distribution of data, continuous variables were presented as mean and standard deviation or as median and interquartile range (IQR). Categorical variables were reported as absolute numbers and percentages. The independent samples t test or the Mann–Whitney U test was used to compare continuous outcome variables as appropriate. Categorical data was tested with a Chi square or Fisher’s exact test. The main unit of analysis was the flap. Because in a bilateral reconstruction both flaps are harvested from the same abdomen, standard comparative analyses cannot be performed as the assumption of independent data is violated. To account for clustered data in patients with a bilateral reconstruction, generalized estimating equations (GEE) with an exchangeable instead of independent structure were used to calculate the association between neoadjuvant chemotherapy and all recipient- and donor-site complications. Both unadjusted and adjusted odds ratios, corrected for clinically relevant variables (i.e., age, follow-up, and reason for mastectomy), were provided with its 95% confidence intervals. *P* values ≤ 0.05 were considered statistically significant. Statistical analyses were conducted using IBM SPSS (version 23.0, SPSS Inc., Chicago, Illinois, USA) for Windows.

## Results

### Patient characteristics

We included 326 eligible patients who underwent a total of 432 immediate DIEP flap breast reconstructions. Forty-eight patients (*n* = 67 flaps) received neoadjuvant chemotherapy prior to immediate breast reconstruction, whereas 278 patients (*n* = 365 flaps) underwent immediate DIEP flap breast reconstruction without neoadjuvant chemotherapy and served as a control group. The patient characteristics are presented in Table 1. Both groups were comparable with respect to BMI, comorbidities, type of reconstruction, and operative time for unilateral and bilateral reconstructions. Patients who received neoadjuvant chemotherapy were significantly younger (46.3 ± 8.1 years vs. 51.3 ± 9.3 years; *p* < 0.001). Significantly more patients in the control group were active smokers (0% vs. 9.4%; *p* = 0.020) at the time of surgery. Median follow-up was 14 months (IQR 7 to 20 months) and 12 months (IQR 7 to 19 months) in the neoadjuvant chemotherapy and control group, respectively (*p* = 0.496).

**Table 1:** Patient characteristics (*n* = 326 patients)

	Neoadjuvant chemotherapy group	Control group	*p* value
	*n* (%)	*n* (%)	
Total number of patients	48	278	
Total number of immediate DIEP flaps	67	365	
Age in years; mean ± SD	46.3 ± 8.1	51.3 ± 9.3	< 0.001
BMI in kg/m^2^; mean ± SD	26.8 ± 3.5	26.7 ± 3.8	0.891
Active smoker	0 (0)	26 (9.4)	0.020^a^
Hypertension	4 (8.3)	47 (16.9)	0.131
Diabetes mellitus	1 (2.1)	14 (5.0)	0.707^a^
Type of reconstruction			
Unilateral	29 (60.4)	191 (68.7)	0.258
Bilateral	19 (39.6)	87 (31.3)	
Operative time in minutes; median (IQR)			
Unilateral breast reconstructions	394 (349–448)	387 (331–450)	0.929
Bilateral breast reconstructions	468 (409–534)	484 (413–574)	0.574
Ischemia time in minutes; median (IQR)^b^	44 (37 –56)	47 (40–60)	0.081
Flap weight in grams; mean ± SD^b^	612 ± 248	645 ± 234	0.304
Hospital stay in days; median (IQR)	7 (6–8)	7 (6–7)	0.908
Follow-up in months; median (IQR)	14 (7–20)	12 (7–19)	0.496

*DIEP* deep inferior epigastric artery perforator, *SD* standard deviation, *BMI* body mass index, *IQR* interquartile range

^a^Fisher’s exact test was used

^b^Total number of flaps as unit of analysis (neoadjuvant chemotherapy group: *n* = 67; control group: *n* = 365)

### Oncological treatment and tumor characteristics

An overview of the oncological treatment of the study population is presented in Table 2. Significantly more patients in the neoadjuvant chemotherapy group underwent mastectomy because of breast cancer compared to the control group in which roughly half of the patients underwent risk-reducing or prophylactic mastectomy (*p* < 0.001). Fifteen patients (22.4%) in the neoadjuvant chemotherapy group underwent contralateral prophylactic mastectomy combined with an immediate bilateral DIEP flap breast reconstruction. Patients in the neoadjuvant chemotherapy group significantly more often received adjuvant endocrine therapy (*p* = 0.007) and immunotherapy (*p* < 0.001), whereas 45 patients in the control group received adjuvant chemotherapy compared to none in the neoadjuvant chemotherapy group (*p* = 0.001). The most frequently administered chemotherapy regimens were triplet chemotherapy consisting of docetaxel, adriamycin, and cyclophosphamide (TAC; 47.9% of patients), and a sequential chemotherapy of Adriamycin and cyclophosphamide followed by a taxane with or without HER2-targeted therapy (35.4% of patients). The time between the last chemotherapy cycle and surgery was a median of 4 weeks (IQR 3 to 6 weeks).

**Table 2:** Oncological treatment (*n* = 326 patients)

	Neoadjuvant chemotherapy group	Control group	*p* value
	*n* (%)	*n* (%)	
Reason for mastectomy^a^			
Oncological	52 (77.6)	182 (49.9)	< 0.001
Prophylactic	15 (22.4)	183 (50.1)	
Genetic predisposition	10 (20.8)	66 (23.7)	0.660
History of lumpectomy^a^	13 (19.4)	64 (17.5)	0.713
Adjuvant chemotherapy	0 (0)	45 (16.2)	0.001^b^
History of radiation therapy^a^	9 (13.4)	55 (15.1)	0.729
Radiation therapy on DIEP flap^a^	2 (3.0)	20 (5.5)	0.552^b^
Endocrine therapy	26 (54.2)	94 (33.8)	0.007
Immunotherapy	18 (37.5)	25 (9.0)	< 0.001
Neoadjuvant chemotherapy regimen			
TAC	23 (47.9)	–	–
ACTH	10 (20.8)	–	–
AC-P	5 (10.4)	–	–
AC-T	2 (4.2)	–	–
Other	8 (16.7)	–	–
Time NAC–surgery in weeks; median (IQR)	4 (3–6)	–	–

*DIEP* deep inferior epigastric artery perforator, *IQR* interquartile range, *TAC* Taxotere (Docetaxel), Adriamycin and Cyclophosphamide, *ACTH* Adriamycin, Cyclophosphamide, Taxol, and Herceptin, *AC*-*P* Adriamycin, Cyclophosphamide, and Taxol (Paclitaxel), *AC*-*T* Adriamycin, Cyclophosphamide, and Taxotere (Docetaxel), *NAC* neoadjuvant chemotherapy

^a^Total number of flaps as unit of analysis (neoadjuvant chemotherapy group: n = 67; control group: n = 365)

^b^Fisher’s exact test was used

The tumor characteristics of all 432 breasts are shown in Table 3. Most patients who received neoadjuvant chemotherapy initially had stage II breast cancer, followed by high-grade stage I or stage III breast cancer. Also, more patients in the neoadjuvant chemotherapy group had triple negative tumors (*n* = 14; 26.9%). In a total of 21 out of 52 breasts (40.4%) a pathological complete response to neoadjuvant chemotherapy was achieved and a near-complete response with a remainder of ductal carcinoma in situ (DCIS) in 3 breasts (5.8%).

**Table 3:** Tumor characteristics (*n* = 432 breasts)

	Neoadjuvant chemotherapy group	Control group
	*n* (%)	*n* (%)
Breast pathology		
No cancer	15 (22.4)	181 (49.6)
DCIS/LCIS	0 (0)	53 (14.5)
Invasive carcinoma	52 (77.6)	129 (35.3)
Other cancer	0 (0)	2 (0.5)
Breast cancer stage/clinical TNM stage^a^		
Stage I	10 (19.2)	63 (48.8)
Stage IIa	31 (59.7)	30 (23.3)
Stage IIb	9 (17.3)	23 (17.8)
Stage IIIa	1 (1.9)	9 (7.0)
Stage IIIb	1 (1.9)	0 (0)
Stage IIIc	0 (0)	1 (0.8)
Stage IV	0 (0)	3 (2.3)
Receptor status^a^		
Estrogen receptor positive	34 (65.4)	102 (79.1)
Progesterone receptor positive	25 (48.1)	85 (65.9)
Her2/Neu positive	18 (34.6)	21 (16.3)
Triple negative	14 (26.9)	15 (11.6)

*DCIS* ductal carcinoma in situ, *LCIS* lobular carcinoma in situ

^a^Total number of invasive carcinomas as unit of analysis (neoadjuvant chemotherapy group: *n* = 52; control group: *n* = 129)

### Recipient-site complications

Major and minor recipient-site complications were observed in 9.5% (41/432 flaps) and 23.4% (101/432 flaps) of flaps, with no significant differences between both groups (Table 4). No statistically significant differences in any of the major or minor postoperative complications were observed between patients who received neoadjuvant chemotherapy and patients with no neoadjuvant chemotherapy prior to immediate DIEP flap breast reconstruction. No total flap losses occurred in the neoadjuvant chemotherapy group versus 9 flap losses in the control group.

**Table 4:** Recipient-site complications (*n* = 432 flaps)

	NAC	Control	OR (95% CI)	*p* value	Adjusted OR (95% CI)^a^	Adjusted *p* value^a^
	(*n* = 67)	(*n* = 365)				
	*n* (%)	*n* (%)				
Major complication (≥ 1)	3 (4.5)	38 (10.4)	0.43 (0.13–1.46)	0.175	0.39 (0.11–1.32)	0.129
Total flap loss	0 (0)	9 (2.5)	–	0.366^b^	–	–
Partial flap loss	2 (3.0)	15 (4.1)	0.78 (0.17–3.58)	0.754	0.73 (0.15–3.70)	0.706
Venous congestion	1 (1.5)	20 (5.5)	0.28 (0.04–2.16)	0.223	0.21 (0.03–1.61)	0.133
Minor complication (≥ 1)	11 (16.4)	90 (24.7)	0.61 (0.30–1.28)	0.191	0.59 (0.28–1.22)	0.151
Infection	3 (4.5)	24 (6.6)	0.70 (0.20–2.41)	0.566	0.69 (0.20–2.42)	0.560
Hematoma	3 (4.5)	34 (9.3)	0.47 (0.14–1.58)	0.221	0.40 (0.12–1.39)	0.149
Seroma	0 (0)	12 (3.3)	–	0.227^b^	–	–
Fat necrosis	4 (6.0)	39 (10.7)	0.53 (0.19–1.52)	0.237	0.51 (0.18–1.49)	0.220
Wound problems	5 (7.5)	35 (9.6)	0.81 (0.27–2.45)	0.714	0.81 (0.27–2.46)	0.709

*NAC* neoadjuvant chemotherapy, *OR* odds ratio, *CI* confidence interval

^a^Adjusted for age (years), follow-up (months), and reason for mastectomy (oncological vs. prophylactic)

^b^Fisher’s exact test was used

The odds ratios of the univariate analyses were adjusted for potential confounding variables (i.e., age in years, follow-up in months, and either oncological or prophylactic reason for mastectomy) in multivariable models. Again, no statistically significant differences in adjusted odds ratios were registered for any of the major or minor complications. Univariate and multivariable analyses of recipient-site complications with the patient instead of the flap as the unit of analysis showed similar results (see additional data “Online Resource 1: Table 1”).

Lastly, in one patient (2.1%) who received neoadjuvant chemotherapy a pulmonary embolism was diagnosed postoperatively, whereas a thromboembolic event was documented in four patients (1.4%; 3 pulmonary embolisms and one deep venous thrombosis) in the control group (unadjusted OR 1.46, 95% CI 0.16–13.3, *p* = 0.739; adjusted OR 2.59, 95% CI 0.20–34.3, *p* = 0.471).

### Donor-site complications

Abdominal herniation as a major donor-site complication was only recorded once (0.4%) in the control group (Table 5). Minor donor-site complications were observed in 9 (18.8%) and 61 (21.9%) patients, respectively, with no significant differences between groups (*p* = 0.619). Of all minor complications, wound healing problems were most frequently documented in both groups (16.7% vs. 16.2%, OR 1.04, 95% CI 0.46–2.36, *p* = 0.934). After adjusting the odds ratios of the univariate analyses for the aforementioned potential confounding variables comparable results were found.

**Table 5:** Donor-site complications (*n* = 326 patients)

	NAC	Control	OR (95% CI)	*p* value	Adjusted OR (95% CI)^a^	Adjusted *p* value^a^
	(*n* = 48)	(*n* = 278)				
	*n* (%)	*n* (%)				
Major complication (≥ 1)	0 (0)	1 (0.4)	–	1.000^b^	–	–
Herniation	0 (0)	1 (0.4)	–	1.000^b^	–	–
Minor complication (≥ 1)	9 (18.8)	61 (21.9)	0.82 (0.38–1.79)	0.619	0.92 (0.40–2.12)	0.841
Infection	4 (8.3)	17 (6.1)	1.40 (0.45–4.34)	0.565	1.84 (0.55–6.18)	0.323
Hematoma	0 (0)	3 (1.1)	–	1.000^b^	–	–
Seroma	0 (0)	10 (3.6)	–	0.368^b^	–	–
Fat necrosis	0 (0)	16 (5.8)	–	0.142^b^	–	–
Bulging	0 (0)	2 (0.7)	–	1.000^b^	–	–
Wound problems	8 (16.7)	45 (16.2)	1.04 (0.46–2.36)	0.934	1.25 (0.51–3.04)	0.628

*NAC* neoadjuvant chemotherapy, *OR* odds ratio, *CI* confidence interval

^a^Adjusted for age (years), follow-up (months), and reason for mastectomy (oncological vs. prophylactic)

^b^Fisher’s exact test was used

### Flap re-explorations

An overview of the flap re-explorations is presented in Table 6. In total, 33 DIEP flaps (7.6%; 2/67 in the neoadjuvant chemotherapy group vs. 31/365 in the control group) required re-exploration and 21 flaps (4.9%; 2/67 in the neoadjuvant chemotherapy group vs. 19/365 in the control group) required reanastomosis of the vein and/or artery, with no significant differences between groups. The most frequent reason for re-exploration of the flap was venous congestion, which was comparable for both groups. Re-exploration of the flap resulted in viable flaps in both cases in the neoadjuvant chemotherapy group and in 61.3% (19/31 flaps) in the control group. Analysis of flap re-explorations with the patient as the unit of analysis also showed similar results (see additional data “Online Resource 1: Table 2”).

**Table 6:** Flap re-explorations (*n* = 432 flaps)

	NAC	Control	*p* value
	(*n* = 67)	(*n* = 365)	
	*n* (%)	*n* (%)	
Re-exploration	2 (3.0)	31 (8.5)	0.139^b^
Reanastomosis	2 (3.0)	19 (5.2)	0.756^b^
Reason re-exploration			
Arterial insufficiency	1 (1.5)	6 (1.6)	1.000^b^
Venous insufficiency	2 (3.0)	21 (5.8)	0.554^b^
Hematoma	0 (0)	7 (1.9)	0.602^b^
Kinking	0 (0)	6 (1.6)	0.596^b^
Result re-exploration^a^			
Viable flap	2 (100.0)	19 (61.3)	0.523^b^
Partial flap loss	0 (0)	3 (9.7)	1.000^b^
Total flap loss	0 (0)	9 (29.0)	1.000^b^

*NAC* neoadjuvant chemotherapy

^a^As a percentage of the total flaps that required re-exploration (NAC group: *n* = 2; control group: *n* = 31)

^b^Fisher’s exact test was used

## Discussion

The aim of this study was to evaluate the influence of neoadjuvant chemotherapy on the complications of immediate DIEP flap breast reconstructions.

Direct comparison of the surgical outcomes of patients with and without neoadjuvant chemotherapy prior to immediate breast reconstruction demonstrated similar complication rates. No statistically significant differences were found between both groups for any major or minor recipient- or donor-site complication, nor for the re-exploration rate of DIEP flap breast reconstructions, also after adjusting for potential confounding variables. Additionally, no delay in adjuvant treatment was documented in the neoadjuvant chemotherapy group.

Our findings are in line with a number of previous studies that showed a similar complication rate irrespective of neoadjuvant chemotherapy use [18–22, 24]. A prospective study by Schaverien et al. [18]. included 22 patients with neoadjuvant chemotherapy and 41 patients without neoadjuvant chemotherapy, who had undergone immediate DIEP flap breast reconstruction. There were no significant differences in postoperative complication rates (67% vs. 65%; *p* = 0.87) or need for reoperations (17% vs. 9%; *p *= 0.30), although three patients in each group had a delay in adjuvant treatment. An interesting point raised by the authors is that the dissection of the perforators seemed to be more difficult in patients who received neoadjuvant chemotherapy. This experience is shared by us, as we feel that chemotherapy might affect the tissue quality and structure, even though we did not find a significant difference in operative time and ischemia time. Zweiffel-Schlatter et al. [20]. also found no significant differences in complication rates (36% vs. 29%) in 47 patients with neoadjuvant chemotherapy compared to 52 patients without neoadjuvant chemotherapy who underwent various types of autologous breast reconstructions. A retrospective study by Azzawi et al. [21]. in a mixed group of patients who underwent either autologous or implant-based breast reconstruction also showed that neoadjuvant chemotherapy did not increase the risk of postoperative complications even though three-quarters of the patients treated with neoadjuvant chemotherapy and a quarter of the controls received adjuvant radiation therapy.

Conversely, some studies reported that neoadjuvant chemotherapy was associated with an increased risk of complications [16, 17]. In a large study by Mehrara et al. [16], 952 patients were included who underwent immediate autologous breast reconstruction, of which 70 patients (7.4%) received neoadjuvant chemotherapy. Neoadjuvant chemotherapy came out as an independent predictor of postoperative complications (OR 2.1; *p* < 0.01) and was associated with (donor-site) wound healing problems (OR 2.9; *p* = 0.02) and fat necrosis (OR 2.8; *p* < 0.01) in multivariate analysis. However, this study did not include DIEP flaps, but mainly transverse rectus abdominis (TRAM) flap breast reconstructions besides other flaps with different complication rates for each type of breast reconstruction. Obesity was a significant predictor of overall complications which is in line with findings of our previous studies [25, 26]. A study by Albino et al. [17]. included patients who underwent immediate autologous breast reconstruction followed by radiation therapy and reported that neoadjuvant chemotherapy increased the incidence of complications (OR 4.4; *p* = 0.04), and more specifically skin complications (OR 2.4; *p* = 0.01).

Surprisingly, a meta-analysis by Song et al. [23]. demonstrated that patients treated with neoadjuvant chemotherapy were even less likely to have complications after immediate breast reconstruction. This conclusion, however, was based on the analysis of a combination of various types of breast reconstruction techniques, ranging from autologous to implant-based reconstruction. No subgroup analysis was done for the specific breast reconstruction techniques, and the results were not adjusted for confounding variables, which makes the results difficult to interpret. Additionally, the meta-analysis did not include the study by Mehrara et al. [16]. that reported an increased risk of complications after neoadjuvant chemotherapy. Also in our study less postoperative complications were observed in patients who received neoadjuvant chemotherapy, even though this trend was not statistically significant, possibly due to the relatively small sample size. Mechanisms behind the protective association of neoadjuvant chemotherapy still remain unknown [23, 27]. In agreement with other authors [19, 23], the selection of patients might have contributed to this observation, as for example patients who received neoadjuvant chemotherapy were significantly younger and more patients in the control group were active smokers.

Limitations of this study include the retrospective study design and the potential bias that are associated with it. Two patient cohorts were constructed which inevitably led to selection bias since patients who had an indication for neoadjuvant chemotherapy initially had a less favorable clinical tumor stage whereas also patients without breast cancer who underwent risk-reducing surgery were included in the control group. Even though this is the largest study to date on specifically DIEP flap breast reconstructions, the sample size is still relatively small and may have influenced the results. No subgroup analysis was performed to assess the effects of time in weeks between the last dose of chemotherapy and surgery. Strengths of this study, however, are the comprehensive analysis of all complications and the multicenter design, which resulted in different oncological treatment plans.

## Conclusions

This study demonstrated similar complication rates for patients with and without neoadjuvant chemotherapy prior to immediate breast reconstruction, indicating that it is safe to perform an immediate DIEP flap breast reconstruction after neoadjuvant chemotherapy. There were no statistically significant differences in the incidences of flap re-explorations, recipient-site complications or donor-site complications. More prospective data is required to confirm these results.

## Electronic supplementary material

Below is the link to the electronic supplementary material.

Supplementary material 1 (DOCX 21 kb)
