# Supplementary material for: The influence of neoadjuvant chemotherapy on complications of immediate DIEP flap breast reconstructions
Source: Breast Cancer Res Treat. 2019 Apr 27;176(2):367–75. doi: 10.1007/s10549-019-05241-9 (PMC6555777; doi:10.1007/s10549-019-05241-9)
Supplement: Supplementary file 1 — Supplementary material 1 (DOCX 21 kb) [file 10549_2019_5241_MOESM1_ESM.docx]

# Online Resource 1: Patient as unit of analysis

**Article title:**

The influence of neoadjuvant chemotherapy on complications of immediate DIEP flap breast reconstructions

**Journal name:**

Breast Cancer Research and Treatment

**Author names:**

J. Beugels^1,2^*, J.L.W. Meijvogel^1^, S.M.H. Tuinder^1^, V.C.G. Tjan-Heijnen^2,3^, E.M. Heuts^4^, A. Piatkowski^1^, and R.R.W.J. van der Hulst^1,2^

**Affiliations:**

^1)^ Department of Plastic, Reconstructive and Hand Surgery, Maastricht University Medical Center, Maastricht, the Netherlands

^2)^ GROW – School for Oncology and Developmental Biology, Maastricht University, Maastricht, the Netherlands

^3)^ Department of Medical Oncology, Maastricht University Medical Center, Maastricht, the Netherlands

^4)^ Department of Surgery, Maastricht University Medical Center, Maastricht, the Netherlands

*** Corresponding author:**

Jop Beugels, *M.D.*

Department of Plastic, Reconstructive and Hand Surgery, Maastricht University Medical Centre, P.O. Box 5800, 6202, AZ, Maastricht, the Netherlands. (+31) 43 387 7481; [jop.beugels@mumc.nl](mailto:jop.beugels@mumc.nl)

| **Table 1. Recipient-site complications (*n* = 326 patients)** | | | | | | |
| --- | --- | --- | --- | --- | --- | --- |
|  | NAC | Control |  |  |  |  |
|  | (*n* = 48) | (*n* = 278) |  |  | Adjusted | Adjusted |
|  | *n* (%) | *n* (%) | OR (95% CI) | *p* value | OR (95% CI) ^a^ | *p* value ^a^ |
| Major complication (≥1) | 3 (6.3) | 36 (12.9) | 0.45 (0.13-1.52) | 0.197 | 0.48 (0.13-1.74) | 0.263 |
| Total flap loss | 0 (0) | 9 (3.2) | - | 0.366 ^b^ | - | - |
| Partial flap loss | 2 (4.2) | 14 (5.0) | 0.82 (0.18-3.73) | 0.797 | 0.94 (0.17-5.09) | 0.939 |
| Venous congestion | 1 (2.1) | 19 (6.8) | 0.29 (0.04-2.22) | 0.233 | 0.25 (0.03-1.95) | 0.187 |
|  |  |  |  |  |  |  |
| Minor complication (≥1) | 10 (20.8) | 81 (29.1) | 0.64 (0.30-1.35) | 0.239 | 0.60 (0.28-1.31) | 0.201 |
| Infection | 3 (6.3) | 22 (7.9) | 0.78 (0.22-2.70) | 0.690 | 0.71 (0.19-2.63) | 0.606 |
| Hematoma | 3 (6.3) | 32 (11.5) | 0.51 (0.15-1.75) | 0.285 | 0.42 (0.12-1.52) | 0.187 |
| Seroma | 0 (0) | 12 (4.3) | - | 0.226 ^b^ | - | - |
| Fat necrosis | 4 (8.3) | 38 (13.7) | 0.57 (0.20-1.69) | 0.314 | 0.71 (0.23-2.20) | 0.546 |
| Wound problems | 4 (8.3) | 32 (11.5) | 0.70 (0.24-2.07) | 0.519 | 0.67 (0.21-2.14) | 0.499 |
| NAC, neoadjuvant chemotherapy; OR, odds ratio; CI, confidence interval  ^a^ Adjusted for age (years), follow-up (months), and reason for mastectomy (oncological vs. prophylactic)  ^b^ Fisher’s exact test was used | | | | | | |

| **Table 2. Flap re-explorations (*n* = 326 patients)** | | | |
| --- | --- | --- | --- |
|  | NAC | Control |  |
|  | (*n* = 48) | (*n* = 278) |  |
|  | *n* (%) | *n* (%) | *p* value |
| Re-exploration | 2 (4.2) | 30 (10.8) | 0.195 ^b^ |
| Reanastomosis | 2 (4.2) | 18 (6.5) | 0.749 ^b^ |
| Reason re-exploration |  |  |  |
| Arterial insufficiency | 1 (2.1) | 6 (2.2) | 1.000 ^b^ |
| Venous insufficiency | 2 (4.2) | 20 (7.2) | 0.754 ^b^ |
| Hematoma | 0 (0) | 7 (2.5) | 0.599 ^b^ |
| Kinking | 0 (0) | 6 (2.2) | 0.598 ^b^ |
| Result re-exploration ^a^ |  |  |  |
| Viable flap | 2 (100.0) | 18 (60.0) | 0.516 ^b^ |
| Partial flap loss | 0 (0) | 3 (10.0) | 1.000 ^b^ |
| Total flap loss | 0 (0) | 9 (30.0) | 1.000 ^b^ |
| ^a^ As a percentage of the total flaps that required re-exploration (NAC group: n = 2; control group: n = 30).  ^b^ Fisher’s exact test was used | | | |
